# The impact of whole-body vibration training on adolescent baseball batting performance

**DOI:** 10.3389/fpsyg.2026.1758424

**Published:** 2026-07-22

**Authors:** Chao-Fu Chen, Lin-Zhu, Teng-Xiang Ge, He-Qian Liu, Hui-Ju Wu, Yan-Qing Shen

**Affiliations:** 1 School of Physical Education and Sport, Henan University, Kaifeng, China; 2 Physical Education College, Zhengzhou University of Industrial Technology, Zhengzhou, China; 3 Physical Education Department, Xiangtan University, Xiangtan, China; 4 Physical Education College, Huaibei Normal University, Huaibei, China; 5 Xiamen Yixue Sports and Technology Co., Ltd., Xiamen, China

**Keywords:** batting mechanics, injury prevention, musculoskeletal health, neuromuscular training, youth baseball

## Abstract

**Introduction:**

Baseball batting is a high-speed, multi-joint coordinated movement that relies on the effective operation of the kinetic chain. Whole-body vibration (WBV) training has been proven to acutely enhance lower-limb muscle strength, but its effects on the batting kinematics of adolescent baseball players remain unclear. This study aimed to investigate the acute effects of WBV training, with and without added load, on the batting kinematics of adolescent baseball players.

**Methods:**

Thirty-two national adolescent baseball players were recruited and randomly divided into four groups: a non-vibration group, a whole-body vibration group, a 30% 1RM WBV group, and a 50% 1RM WBV group. All four groups performed the training in a semi-squat calf-raise posture for 60 s. Immediately after the intervention, participants performed maximal-effort swings, which were analyzed using the Kwon3D motion capture system.

**Results:**

When comparing the four conditions, both the non-loaded WBV and loaded WBV groups exhibited higher lower-limb and bat head speeds, with overall performance superior to the non-vibration group. The 50% 1RM group showed the highest knee joint angular velocity, while the 30% 1RM group was most prominent in rotation-related indicators for the thigh and pelvis segments. The non-loaded WBV group showed the highest elbow joint angular velocity. At the moment of bat-ball contact (mid-batting instant), the pelvic linear velocity in the WBV and 30% 1RM groups was significantly greater than that in the non-vibration group.

**Conclusion:**

All WBV training protocols acutely optimized key segments of the kinetic chain in the batting swing of adolescent baseball players. This offers a time-efficient training strategy and can serve as a practical reference for pre-training or pre-competition preparation.

## 1 Introduction

Baseball bat swing is a high-speed, explosive movement involving a kinetic chain, which requires the efficient transmission of force and energy among the lower extremities, pelvis, torso, upper extremities, and the bat itself (Orishimo et al., 2024). Key factors influencing swing performance include: lower body force generation and the utilization of ground reaction force (GRF), parameters such as hip-shoulder separation, counter-movement, backswing, torso rotation speed, and the timing of the upper extremities and wrists (Semkewyc and Craelius, 2023). At the outcome level, commonly used assessment metrics include bat velocity, exit velocity, and time to contact (Kohn et al., 2024). In the field of sports medicine and performance optimization, effective dynamic warm-ups can improve muscle blood flow, enhance nerve conduction and muscle contraction speed, increase joint range of motion (ROM), and reduce injury risk, thereby creating optimal neuromuscular conditions for high-speed swinging (Balan et al., 2024).

In recent years, Whole-Body Vibration (WBV) training has been widely applied in strength and conditioning (Sharma and Hayat, 2022). WBV can reinforce reflexes during muscle contraction, inducing the Tonic Vibration Reflex (TVR), which subsequently affects muscle spindle proprioception and alters the length regulation and contraction speed of skeletal muscle fibers (Kalaoglu et al., 2023). Previous studies have shown that implementing WBV with stable vibration intensity and protocols can promote muscle adaptation and enhance multiple neuromuscular and physical fitness indicators, including muscle strength and explosiveness, flexibility, body composition, and Delayed Onset Muscle Soreness (DOMS) (Bonanni et al., 2022). Therefore, incorporating vibration stimulation into a training program helps optimize muscle contraction characteristics and motor output. The positive short-term effects of WBV on lower limb explosiveness have been validated by multiple experiments; for instance, protocols using a frequency of 30 Hz are commonly seen in interventions aimed at enhancing explosiveness. Under various combinations of frequencies (30 to 50 Hz) and amplitudes (2 to 5 mm), the immediate effects of WBV are typically most pronounced immediately after the intervention up to 1 min, and may attenuate after approximately 5 min. All of the above suggest that an appropriate WBV protocol can serve as an acute activation method before sport-specific technical training (Wang et al., 2022; Haleva et al., 2023; Usgu and Yüksel, 2022).

Furthermore, as a complex kinetic chain movement, baseball bat swing performance is not only dependent on the generation of lower limb explosiveness and ground reaction force (GRF) but also relies on the efficient transmission and synergistic output of energy between the torso and upper extremities (Ahmad and Schulz, 2021). However, current empirical evidence regarding the inclusion of Whole-Body Vibration (WBV) training in baseball bat swing protocols and the examination of its training effects on improving key technical parameters and kinetic chain efficiency remains relatively limited. Existing research predominantly focuses on general physical fitness items such as jumping, squatting, or lower body strength, with a relative lack of systematic discussion on utilizing WBV as a specific intervention to enhance baseball-specific technical performance (AlBaiti et al., 2024). In addition, the existing literature review is often not sufficiently systematic in explaining the potential application mechanisms of whole-body vibration training (WBV) for baseball batting (e.g., neuromuscular activation, enhanced proprioceptive input, post-activation potentiation–related effects, and improved inter-segmental coordination), which makes it difficult to establish a clear mechanistic pathway from (WBV) stimulus to batting-specific outcomes (Orishimo et al., 2024; Semkewyc and Craelius, 2023; Kohn et al., 2024; Balan et al., 2024; Sharma and Hayat, 2022; Kalaoglu et al., 2023; Bonanni et al., 2022; Wang et al., 2022; Haleva et al., 2023; Usgu and Yüksel, 2022; Ahmad and Schulz, 2021; AlBaiti et al., 2024). Moreover, the rationale for selecting 30% 1RM and 50% 1RM as external load gradients in WBV-based protocols has not been clearly articulated in prior work, and the theoretical basis for these specific intensities (e.g., balancing neuromuscular facilitation versus movement quality/fatigue, or aligning with commonly used “light-to-moderate” resistance ranges for power-oriented activation) remains insufficiently justified (Orishimo et al., 2024; Semkewyc and Craelius, 2023; Kohn et al., 2024; Balan et al., 2024; Sharma and Hayat, 2022; Kalaoglu et al., 2023; Bonanni et al., 2022; Wang et al., 2022; Haleva et al., 2023; Usgu and Yüksel, 2022; Ahmad and Schulz, 2021; AlBaiti et al., 2024).

Therefore, this study aims to use WBV as an intervention training method for baseball bat swing and compare the differences in athletic performance among four key technical phases—foot lift instant, center of gravity zero point instant, hip rotation instant, and bat-ball contact instant—across the following groups: the Non-Vibration Group, the Vibration Group, the 30% 1RM Vibration Group, and the 50% 1RM Vibration Group. This comparison seeks to reveal the effects of different vibration protocols on bat swing motor output and the synergistic efficiency of the kinetic chain.

## 2 Materials and methods

### 2.1 Participants

This study recruited a total of 32 national-level adolescent male baseball players, with an average age of 15.8 ± 0.6 years, average height of 179.3 ± 5.4 cm, and average weight of 70.8 ± 6.9 kg. The participants were randomly divided into four groups: Non-Vibration Group (*n* = 8), Vibration Group (*n* = 8), 30% 1RM Vibration Group (*n* = 8), and 50% 1RM Vibration Group (*n* = 8). All testing movements were completed by the participants using their dominant hand. To examine the consistency of baseline characteristics among the groups, a one-way analysis of variance (ANOVA) was used to compare variables such as age, height, weight, BMI, and years of sports participation. The results showed no significant differences among the groups for all variables (*p* > 0.05), indicating good comparability between the groups. This study was approved by the local university’s Academic Ethics Committee. All experimental procedures adhered to the ethical guidelines of the 1964 Declaration of Helsinki and its subsequent amendments, and written informed consent was obtained from the participants and their legal guardians.

### 2.2 Experimental procedure

This study employed a randomized controlled design to examine the immediate effects of different vibration conditions and loading levels on batting performance. Participants were randomly assigned to one of the four experimental conditions. All tests were performed using the participant’s dominant hand. The standardized procedure was as follows: Participants first performed a 60s partial squat heel raise, immediately followed by three batting tests. The performance from the swing with the highest ball velocity was used to evaluate the immediate intervention effect. Furthermore, the partial squat heel raise was standardized to a knee flexion angle of approximately 120°. Existing research suggests that this angle is beneficial for enhancing neuromuscular activation and force transmission efficiency in the lower limbs and torso, thereby optimizing performance in short-duration explosive tasks (Orishimo et al., 2024).

**Figure 1:**
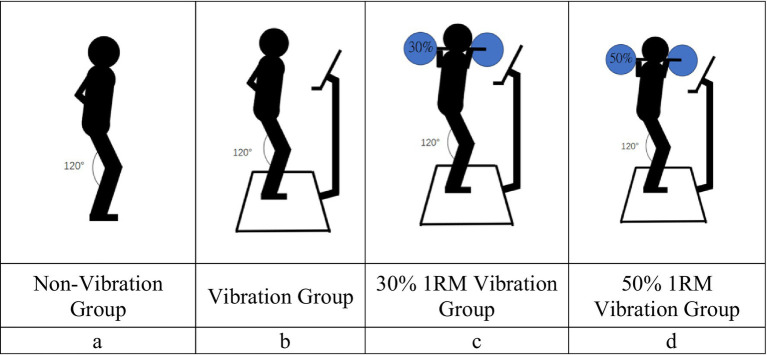
**(a)** Non-vibration group. **(b)** Vibration group. **(c)** 30% 1RM vibration group. **(d)** 50% 1RM vibration group.

- Non-Vibration Group: Participants completed the partial squat heel raise on the ground for 60 s, immediately followed by 3 batting swings. The performance data from the swing with the highest ball velocity out of the three attempts was recorded (Figure 1a).
- Vibration Group: Participants performed the partial squat heel raise for 60 s at 50 Hz and 2 mm (displacement amplitude), immediately followed by 3 batting swings. The performance data from the swing with the highest ball velocity was recorded (Figure 1b).
- 30% 1RM Vibration Group: Under the condition of 50 Hz and 2 mm (displacement amplitude), participants performed the partial squat heel raise for 60 s while bearing a load equivalent to 30% of their individual deep squat 1RM. This was immediately followed by 3 batting swings, and the performance data from the swing with the highest ball velocity was recorded (Figure 1c).
- 50% 1RM Vibration Group: Under the condition of 50 Hz and 2 mm (displacement amplitude), participants completed the partial squat heel raise for 60 s while bearing a load equivalent to 50% of their individual deep squat 1RM. This was immediately followed by 3 batting swings, and the performance data from the swing with the highest ball velocity was recorded (Figure 1d).

**Figure 2:**
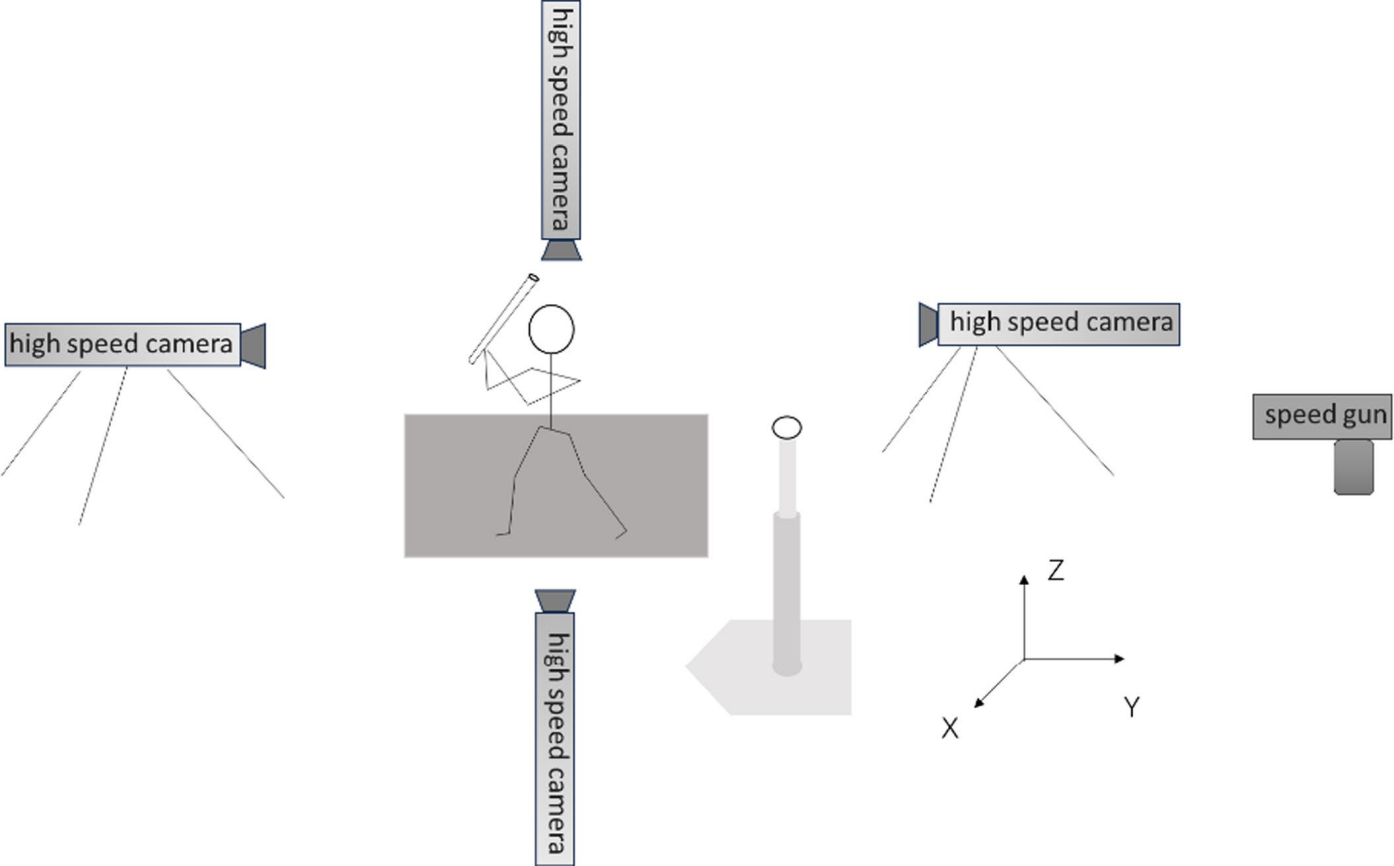
Experimental site setup.

Prior to the formal experiment, this study completed the setup and calibration of the site and equipment. First, a rectangular hitting zone (batter’s box) was marked out on both the left and right sides of the home plate (Tee stand). The dimensions of each zone were 1.82 m long and 1.22 m wide, with the inner edge of each zone located 0.15 m from the edge of the home plate. A spatial coordinate frame (2 m x 1.5 m x 2 m) was installed along the outer perimeter of the hitting zones to establish the laboratory coordinate system. The coordinate system was defined as follows: the initial facing direction of the subject was the positive y-axis, the subject’s left side was the positive x-axis, and the vertical upward direction was the positive z-axis. Stereoscopic calibration was then performed using 12 known coordinate points (AlBaiti et al., 2024; Chen C. F. et al., 2025d; Chen C. F. et al., 2025c; Chen C. et al., 2025). The subjects used their dominant hand for batting. A 14-segment human body model was established, and reflective markers were affixed to the key anatomical landmarks. High-speed cameras were positioned 4 m away, both directly in front of and to the side of the geometric center of the coordinate frame. The camera recording frequency was set to 240 Hz, the shutter speed was set to 1/1000 s, and white balance, exposure settings, and time synchronization were completed before formal data acquisition. The speed radar gun was placed on the extension line of the hitting direction, approximately at the same height as the batting plane, while avoiding the flight path of the bat and the ball (Chen et al., 2024; Chen et al., 2023; Chen et al., 2022; Chen C. F. et al., 2025a, 2025b). The camera system acquired and stored data via a desktop computer (Figure 2). Each athlete performed three maximal-effort swings to hit the ball. The data from the swing that resulted in the highest batted ball exit velocity was selected for subsequent analysis. Finally, the recorded images were digitized and kinematic parameters were calculated using the Kwon3D motion analysis system.

### 2.3 Definition of technology phase division

**Figure 3:**
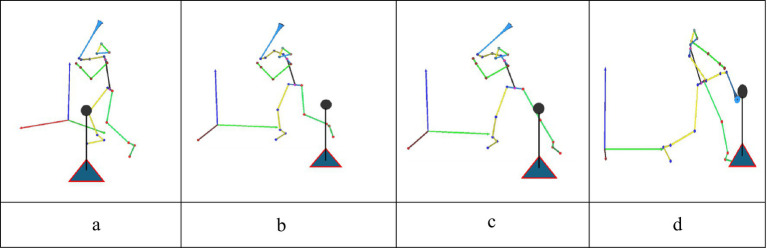
Division of technical stages. **(a)** Foot lift moment. **(b)** Center of gravity zero moment. **(c)** Hip rotation moment. **(d)** Impact moment.

Based on the characteristics of the batting technique, this study divided the movement process into key moments and phases to facilitate parameter extraction and comparative analysis (Figure 3) (Chen et al., 2023; Chen et al., 2022; Chen C. F. et al., 2025a; Chen and Wu, 2022; Chen et al., 2021). Among these, the Foot Lift Moment was defined as the instant when the velocity of the athlete’s left heel bone drops to zero; the Center of Gravity Zero Moment was the instant when the linear velocity of the right hip joint was zero; the Hip Rotation Moment was the time point when the right hip joint begins to accelerate its rotation; and the impact Moment was the instant when the bat makes contact with the ball.

Surrounding these key moments, three continuous motion phases were further delineated:

- Body load phase (wind-up): refers to the process of storing potential energy by slightly shifting the upper body backward and pulling the hands back simultaneously with the foot lift.
- Hip initiation phase: is the process of the hip joint explosively rotating inward, transferring the power generated by the lower limbs toward the torso and upper limbs.
- Impact phase: is the critical moment when the bat strikes the incoming ball, and the entire body’s kinetic energy is delivered through the end of the swing.

Through the definition of these moments and phases, the timing and output characteristics of the kinetic chain during the swing can be systematically evaluated.

This study employed a 4th-order Butterworth low-pass filter to smooth the raw three-dimensional motion data. The cutoff frequency was set to 6 Hz for the calculation of various kinematic parameters. This data processing procedure effectively improved the accuracy and stability of the kinematic parameters, thereby enhancing the overall data quality and strengthening the credibility of the analysis results.

### 2.4 Statistical analysis

This study used a one-way analysis of variance to compare the differences in various kinematic and kinetic parameters among the no vibration group, the vibration group, the 30% 1RM vibration group, and the 50% 1RM vibration group. The level of significance was set at *α* = 0.05.

## 3 Results

**Figure 4:**
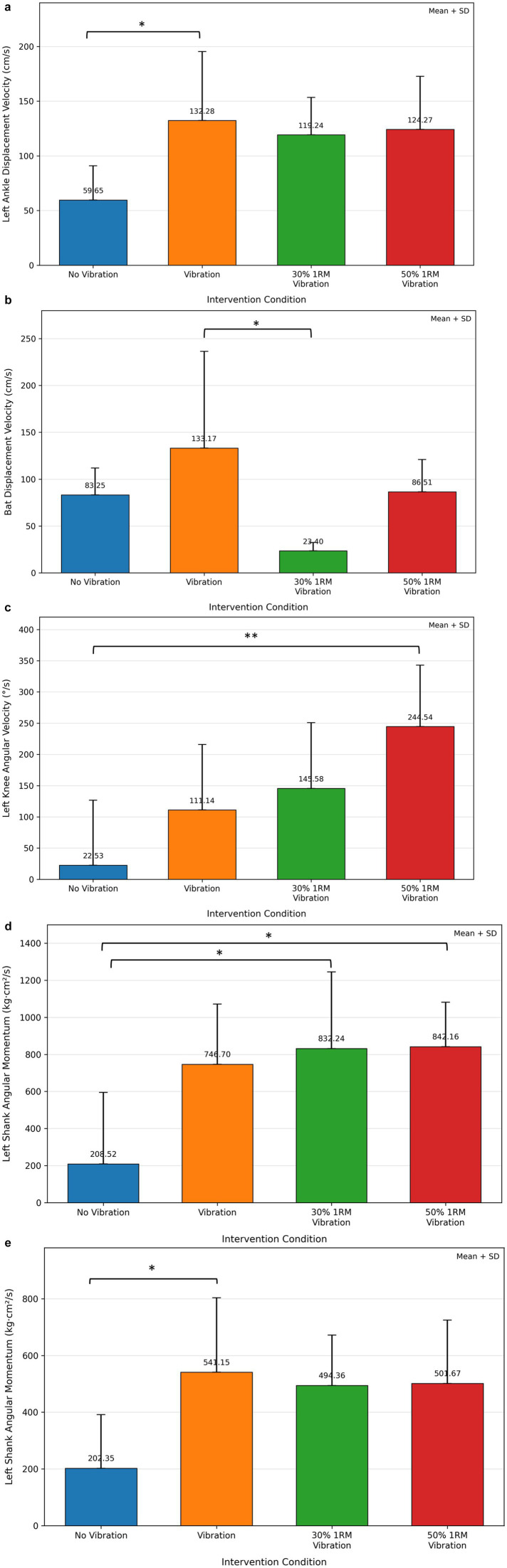
Displacement velocity, joint angular velocity, and angular momentum of key movements at the foot lift moment. **(a)** Left ankle displacement velocity. **(b)** Bat displacement velocity. **(c)** Left knee angular velocity (angular velocity, °/s). **(d)** Left shank angular momentum (around Z-axis). **(e)** Left shank angular momentum (around X-axis).

At the foot lift moment, there were significant differences across the four groups for several parameters:

A  Left ankle displacement velocity:

- No vibration group (group 1): 59.65 ± 31.23 cm/s
- Vibration Group (Group 2): 132.28 ± 63.10 cm/s
- 30%1RM Vibration Group (Group 3): 119.24 ± 34.26 cm/s
- 50%1RM Vibration Group (Group 4): 124.27 ± 48.48 cm/s
- A significant difference was found (*F* = 3.54, *p* = 0.03). Post-hoc analysis showed Group 2 > 1 (Cohen’s d = 1.46, large effect) (Figure 4a).

B  Bat displacement velocity:

- No Vibration Group: 83.25 ± 28.66 cm/s
- Vibration Group: 133.17 ± 103.30 cm/s
- 30%1RM Vibration Group: 23.40 ± 8.97 cm/s
- 50% 1RM Vibration Group: 86.51 ± 34.42 cm/s
- A significant difference was found (*F* = 3.80, *p* = 0.02). Post-hoc analysis showed Group 2 > 3 (Cohen’s d = 1.50, large effect) (Figure 4b).

C  Left knee angular velocity:

- No Vibration Group: 22.53 ± 104.10 ^o^/s
- Vibration Group: 111.14 ± 104.69 ^o^/s
- 30% 1RM Vibration Group: 145.58 ± 105.12 ^o^/s
- 50% 1RM Vibration Group: 244.54 ± 98.31 ^o^/s

A significant difference was found (*F* = 5.10, *p* = 0.00). Post-hoc analysis showed Group 4 > 1 (Cohen’s d = 2.19, large effect) (Figure 4c).

D  Left shank angular momentum (around Z-axis):

- No vibration group: 208.52 ± 386.81 kg‧cm^2^/s
- Vibration group: 746.70 ± 325.35 kg‧cm^2^/s
- 30% 1RM vibration group: $832.24 ± 413.24 kg‧cm^2^/s
- 50% 1RM vibration group: $842.16 ± 239.17 kg‧cm^2^/s
- A significant difference was found (*F* = 5.08, *p* = 0.01). Post-hoc analysis showed Groups 3 and 4 were greater than Group 1 (Group 3 vs. 1: Cohen’s d = 1.56, large; Group 4 vs. 1: Cohen’s d = 1.97, very large), while no difference was observed between Groups 3 and 4 (Figure 4d).

E  Left shank angular momentum (around X-axis):

- No Vibration Group: 202.35 ± 189.58 kg‧cm^2^/s
- Vibration Group: 541.15 ± 262.58 kg‧cm^2^/s
- 30% 1RM Vibration Group: 494.36 ± 177.91 kg‧cm^2^/s
- 50% 1RM Vibration Group: 501.67 ± 223.31 kg‧cm^2^/s
- A significant difference was found (*F* = 3.57, *p* = 0.03). Post-hoc analysis showed Group 2 > 1 (Cohen’s d = 1.48, large effect) (Figure 4e).

**Figure 5:**
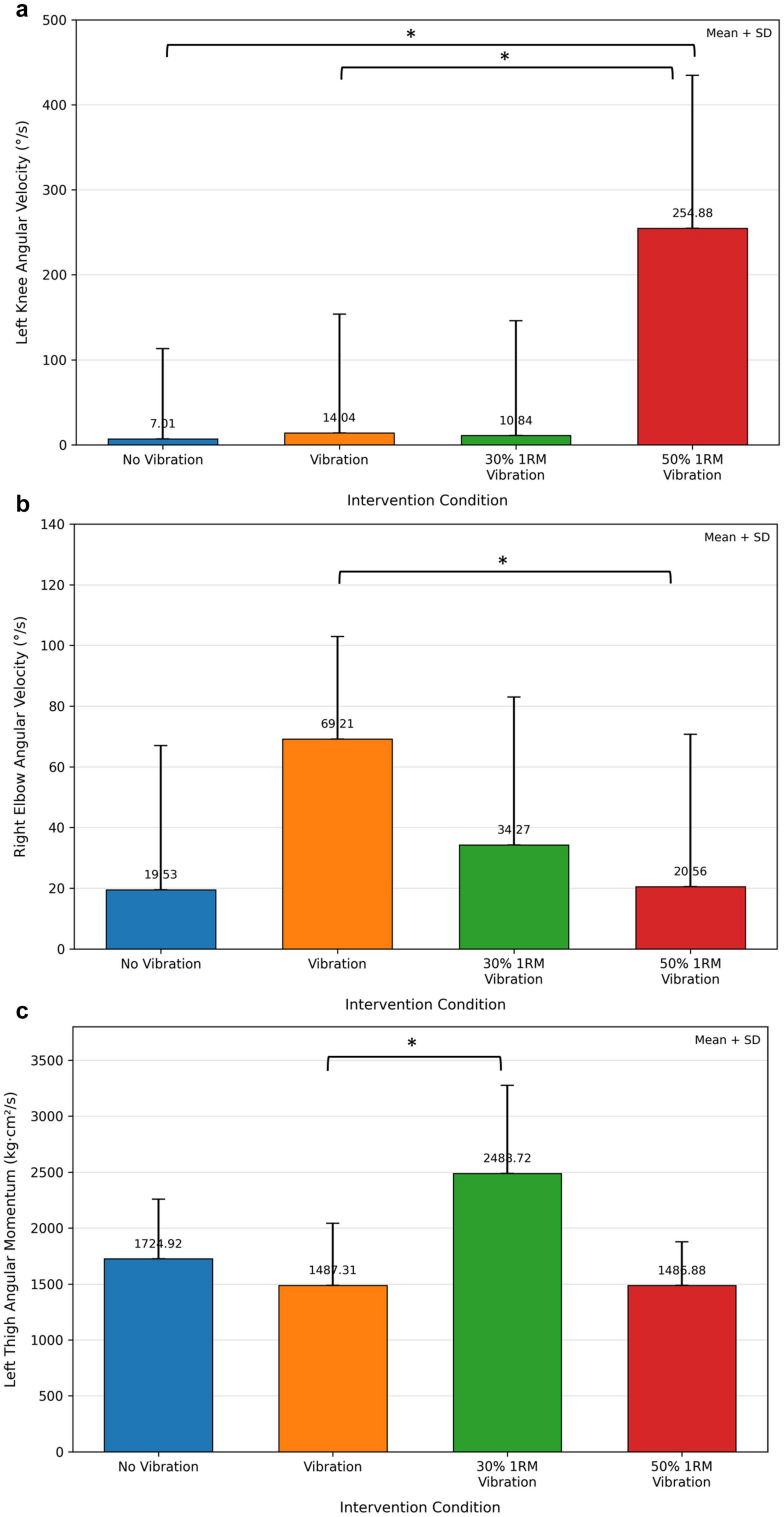
Joint angular velocity and angular momentum of key movements at the hip rotation moment. **(a)** Left knee angular velocity (angular velocity, °/s). **(b)** Right elbow angular velocity (angular velocity, °/s). **(c)** Left thigh angular momentum (around Z-axis).

At the hip rotation moment, there were significant differences across the four groups for the following parameters:

A  Left knee angular velocity:

- No vibration group: 7.01 ± 106.04 o/s
- Vibration group: 14.04 ± 139.77 o/s
- 30% 1RM vibration group: 10.84 ± 135.11 o/s
- 50% 1RM vibration group: 254.88 ± 179.95 ^o^/s
- A significant difference was found (*F* = 4.61, *p* = 0.01). Post-hoc analysis showed Group 4 > Groups 1 and 2 (Group 4 vs. 1: Cohen’s d = 1.68, large; Group 4 vs. 2: Cohen’s d = 1.49, large), while no difference was observed between Groups 1 and 2 (Figure 5a).

B  Right elbow angular velocity:

- No vibration group: 19.53 ± 47.41 ^o^/s
- Vibration group: 69.21 ± 33.70 ^o^/s
- 30% 1RM vibration group: 34.27 ± 48.76 ^o^/s
- 50% 1RM vibration group: 20.56 ± 50.19 ^o^/s
- A significant difference was found (*F* = 4.38, *p* = 0.01). Post-hoc analysis showed Group 2 > Group 4 (Cohen’s d = 1.14, large) (Figure 5b).

C  Left thigh angular momentum (around Z-axis):

- No vibration group: 1724.92 ± 534.63 kg‧cm^2^/s
- Vibration group: 1487.31 ± 556.38 kg‧cm^2^/s
- 30% 1RM vibration group: 2488.72 ± 785.82 kg‧cm^2^/s
- 50% 1RM vibration group: 1486.88 ± 390.72 kg‧cm^2^/s

A significant difference was found (*F* = 4.10, *p* = 0.02). Post-hoc analysis showed Group 3 > Group 2 (Cohen’s d = 1.47, large) (Figure 5c).

**Figure 6:**
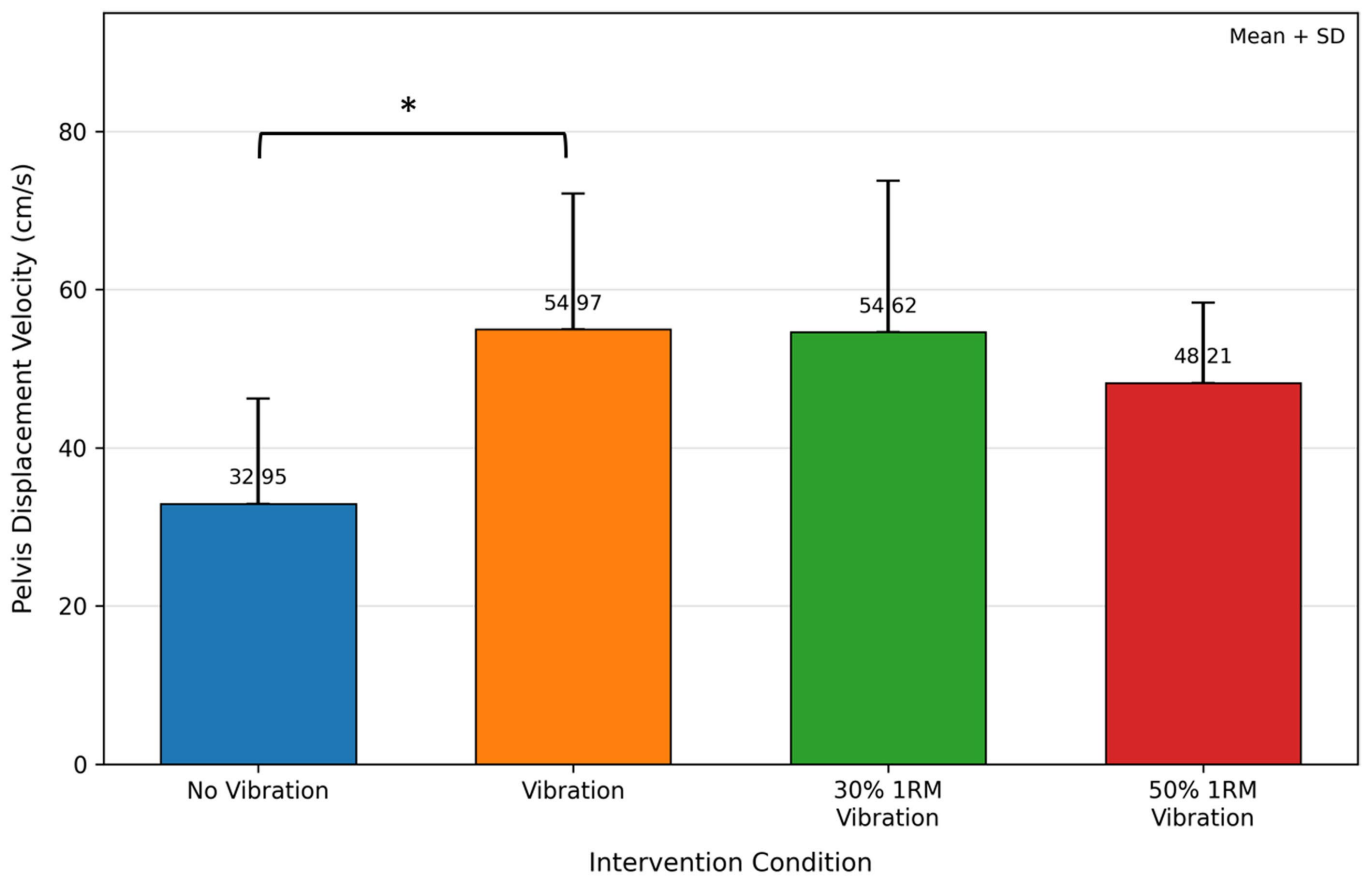
Pelvis displacement velocity at the impact moment.

At the impact moment, the pelvis displacement velocity showed a significant difference across the groups:

- No Vibration Group: 32.95 ± 13.28 cm/s
- Vibration Group: 54.97 ± 17.17 cm/s
- 30% 1RM Vibration Group: 54.62 ± 19.13 cm/s
- 50% 1RM Vibration Group: 48.21 ± 10.15 cm/s
- A significant difference was found (*F* = 3.09, *p* = 0.03). Post-hoc analysis showed Group 2 > Group 1 (Cohen’s d = 1.43, large effect) (Figure 6).

## 4 Discussion

The purpose of this study was to compare the kinematic and kinetic differences in four key technical moments of a baseball swing under different vibration conditions: No Vibration, Whole-Body Vibration (WBV), 30% 1RM Whole-Body Vibration, and 50% 1RM Whole-Body Vibration. Furthermore, the study aimed to verify whether WBV training, either alone or combined with loading, could enhance kinetic chain output capability. The overall results indicate that WBV training significantly improved the motion performance of the lower limbs and torso across multiple movement moments. This effect was most pronounced during the Foot Lift Moment (the preparation and loading phase) and the Hip Rotation Moment (the kinetic chain initiation phase), suggesting that WBV training makes a critical contribution to energy generation during the early and middle stages of the batting movement chain.

The Foot Lift Moment reflects the batter’s pre-stretch preparation and kinetic chain pre-loading state (Goecking et al., 2024). The results of this study indicate that the Vibration Group and the Loaded Vibration Groups 30% 1RM and 50% 1RM showed significantly higher values than the No Vibration Group in indicators such as left ankle velocity, bat velocity, knee angular velocity, and shank angular momentum. Notably, the 50% 1RM group was the most prominent in terms of knee angular velocity and shank angular momentum. This finding suggests that Whole-Body Vibration (WBV) training may help athletes more effectively enter the pre-load state (where muscles are lengthened before being shortened) by increasing muscle spindle sensitivity and enhancing $\alpha$ motor neuron activation. This enables the lower limbs to generate a faster stretch-shortening cycle (SSC) response, thereby improving the kinetic reserve at the initiation phase of the swing (Cetin et al., 2025; Santuz and Akay, 2023; Groeber et al., 2021). Furthermore, the superposition of vibration stimulus and loading can enhance central nervous system (CNS) drive and increase the recruitment ratio of fast-twitch muscle fibers. This is beneficial for strengthening the synchronized pre-activation of the hip, knee, and ankle joints, thereby accelerating the initiation process of the kinetic chain in the swing (Guilherme et al., 2022; Lloyd et al., 2023; Adeel et al., 2024). The Hip Rotation Moment is the core link for the transmission of kinetic chain energy from the lower limbs to the torso (Shafei and Sadeghi, 2022). The results of this study indicated that the Left Knee Angular Velocity under the 50% 1RM Whole-Body Vibration condition was significantly higher than both the No Vibration Group and the Single Vibration Group, suggesting that a higher load is more effective in stimulating the explosive output of the proximal joints. Concurrently, the 30% 1RM Whole-Body Vibration Group showed the best performance in Thigh Angular Momentum, illustrating that a moderate load is more conducive to generating rotational energy in the mid-section of the kinetic chain (thigh and pelvis region). Conversely, the Single Vibration Group recorded the highest Elbow Angular Velocity, reflecting a quicker energy transfer and acceleration at the distal upper extremity under a lower mechanical load. Its movement rhythm is, therefore, more inclined toward speed-type rather than power-type (Colomar et al., 2022; Rigozzi et al., 2023). These findings also indirectly support the view that differences in loading alter the kinetic chain’s energy distribution strategy: higher loads favor power output, while lower loads accentuate speed performance. At the Impact Moment, the Pelvis Velocity in both the Vibration Group and the 30% 1RM Vibration Group was significantly higher than in the No Vibration Group. This demonstrates that Whole-Body Vibration (WBV) training not only helps improve lower limb force generation but also optimizes torso rotation efficiency, enabling the pelvis to reach the ideal hitting position more quickly. This enhancement in pelvic rotation efficiency directly correlates with higher batted ball exit velocity and swing speed (Monteiro et al., 2017; Kapeleti et al., 2024). Previous research indicates that batting performance highly relies on the synergistic effect between proximal acceleration and distal gain. The finding that WBV significantly enhances the rotational capability of the pelvis and torso during the proximal phase suggests that WBV makes a profound contribution to energy transfer within the baseball kinetic chain (Crotin et al., 2022).

The acute effects of Whole-Body Vibration (WBV) typically peak rapidly shortly after the intervention concludes. In this study, the batting tests were scheduled immediately following the intervention to ensure that the results fully reflected the immediate gain effect of WBV (Cochrane et al., 2010; Wei et al., 2025; Garbisu-Hualde and Santos-Concejero, 2021). Furthermore, the results of this study have practical significance. For youth baseball athletes, whose neuromuscular systems are still in the developmental phase, WBV offers a training modality that significantly enhances neuromuscular activation without imposing high joint stress. Specifically, when integrated into pre-game warm-ups and skill training, incorporating WBV into the technical preparation phase may help athletes more rapidly enter their optimal swing state, thereby improving hitting quality (Olsen et al., 2025).

In summary, this study demonstrates that Whole-Body Vibration (WBV) exerts a positive facilitative effect across multiple phases of the baseball swing. Moreover, WBV at different loading levels produces segment-specific gains within the kinetic chain, suggesting that WBV not only influences muscle strength and explosiveness but also modulates inter-segmental coordination and energy transfer. Therefore, WBV may serve as a practical strategy for inclusion in baseball-specific training systems. Nevertheless, several limitations should be acknowledged. In particular, this study evaluated only acute responses; thus, the observed benefits may not necessarily translate to long-term training adaptations.

## 5 Conclusion

This study compared baseball swing mechanics across four vibration conditions and showed that WBV can acutely enhance the mechanical output of multiple segments within the kinetic chain. Beyond reporting isolated variable changes, the key scientific insight is that WBV appears to act as a neuromuscular “priming” stimulus that preferentially strengthens proximal initiation and trunk–pelvis rotational transmission in a proximal-to-distal striking task, thereby improving the readiness and coordination of the kinetic chain during early-to-mid swing phases. Moreover, the results suggest a load-dependent modulation pattern: adding external load during WBV may shift the kinetic-chain strategy toward greater proximal power expression, whereas WBV alone may facilitate faster distal timing/acceleration. Collectively, these findings support the concept that WBV influences not only strength or explosiveness, but also the inter-segmental coordination and energy-transfer efficiency that underpin baseball-specific swing performance.

From an applied perspective, the present data support WBV as a short-duration pre-activation option to acutely prime swing mechanics, but prescriptions should be individualized and integrated with training cycles. Specifically, loaded WBV (30–50% 1RM) may be considered when the training objective is to emphasize proximal drive and rotational power, whereas unloaded WBV may be more appropriate when emphasizing movement timing and speed-oriented coordination. Importantly, these recommendations should be interpreted as acute, context-dependent tendencies rather than universal rules, as responses may vary with athlete maturity, strength level, fatigue state, and technical proficiency.

In summary, WBV, especially when combined with appropriate loads of 30% 1RM and 50% 1RM, is an effective training intervention for improving the immediate performance of a baseball swing. In practical applications:

- 50%1RM is suitable for strengthening lower limb explosiveness and hip rotation speed.
- 30% 1RM helps optimize the rotational efficiency of the pelvis and thigh segments.
- Pure WBV training is useful for improving overall swing speed and timing coordination, and can be used as a short-duration acute muscle activation method before training or competition to induce Post-Activation Potentiation (PAP), serving as a preparation strategy for immediate gain.

## Data Availability

The original contributions presented in the study are included in the article/supplementary material, further inquiries can be directed to the corresponding author.
